# Pathogenic Role of Epstein–Barr Virus in Lung Cancers

**DOI:** 10.3390/v13050877

**Published:** 2021-05-11

**Authors:** David Becnel, Ramsy Abdelghani, Asuka Nanbo, Janardhan Avilala, Jacob Kahn, Li Li, Zhen Lin

**Affiliations:** 1 Department of Medicine, Tulane University Health Sciences Center, New Orleans, LA 70112, USA; dbecnel1@tulane.edu (D.B.); rabdelgh@tulane.edu (R.A.); 2 National Research Center for the Control and Prevention of Infectious Diseases, Nagasaki University, Sakamoto, Nagasaki 852-8523, Japan; nanboa@nagasaki-u.ac.jp; 3 Department of Pathology and Laboratory Medicine, Tulane University Health Sciences Center and Tulane Cancer Center, New Orleans, LA 70112, USA; javilala@tulane.edu (J.A.); jkahn1@tulane.edu (J.K.); 4 Institute of Translational Research, Ochsner Clinic Foundation, New Orleans, LA 70121, USA; lli@ochsner.org

**Keywords:** non-small cell lung cancer, NSCLC, small cell lung cancer, SCLC, Epstein–Barr virus, EBV, next-generation sequencing, NGS

## Abstract

Human oncogenic viruses account for at least 12% of total cancer cases worldwide. Epstein–Barr virus (EBV) is the first identified human oncogenic virus and it alone causes ~200,000 cancer cases and ~1.8% of total cancer-related death annually. Over the past 40 years, increasing lines of evidence have supported a causal link between EBV infection and a subgroup of lung cancers (LCs). In this article, we review the current understanding of the EBV-LC association and the etiological role of EBV in lung carcinogenesis. We also discuss the clinical impact of the knowledge gained from previous research, challenges, and future directions in this field. Given the high clinical relevance of EBV-LC association, there is an urgent need for further investigation on this topic.

## 1. Lung Cancers

Lung cancers (LCs) are the number one killer among cancers in the U.S. and estimated to cause more than 131,000 deaths in 2021 [1]. With more than 200,000 annual cases in the U.S., LCs have remained as the second most common cancers in both men and women for many years [1]. Based on the histological features, LCs can be classified as small cell lung cancers (SCLCs) and non-small cell lung cancers (NSCLCs) according to the WHO 2015 guidelines [2]. NSCLCs account for ~85% of total LC cases and have two major histological sub-types (adenocarcinoma (LUAD) and squamous cell carcinoma (LUSC)) as well as several other rarer sub-types, including pulmonary lymphoepithelioma-like carcinoma (LELC) [2,3].

LC is a complex and multifactorial malignant disease with a currently unclear etiology. It is believed that genetic, epigenetic, and environmental factors all can contribute to lung cancer development. Among those known risk factors, smoking is a definitive oncogenic factor for LC development. Nevertheless, the incidence of LCs is only slowly declining even after the dramatic reduction of smoking through public health awareness campaigns. To date, only a small portion (10–20%) of smokers develop LCs [4]. With a lower smoking rate seen among females, it is estimated that at least 50% of female LC patients worldwide are never smokers. In addition, LCs are the seventh most common cause of cancer death in never smokers [5,6]. Together, this evidence indicates that other major etiological factors are responsible for LC development. The concept of infectious agents as a potential oncogenic trigger for the LC development has long been proposed. However, a causal link between most of microbial infections and LCs has not yet been established.

## 2. Epstein–Barr Virus

Epstein–Barr virus (EBV) is also known as human herpesvirus 4 and belongs to the gamma-herpesvirus subfamily, in the same family with Kaposi’s sarcoma-associated herpesvirus (KSHV). EBV is the first human oncogenic virus discovered in 1964, which currently accounts for ~200,000 annual cancer cases and ~1.8% of all cancer-related deaths globally [7,8,9,10,11]. EBV is etiologically associated with a number of human lymphoid and epithelial malignancies including Burkitt’s lymphoma (BL), Hodgkin’s disease (HD), extranodal nasal-type natural killer/T cell lymphoma (NKTL), post-transplant lymphoproliferative disease (PTLD) and lymphomas (e.g., diffuse large B-cell lymphoma) in immunocompromised individuals, nasopharyngeal carcinoma (NPC), gastric carcinoma (GC), as well as a subset of lung cancers (LCs) [7,8,9,12,13,14,15].

Like other herpesviruses, EBV employs two distinct stages to complete its life cycle: latency and lytic cycle (or lytic reactivation). After an initial, usually asymptomatic infection, EBV will establish a life-long persistent infection in the host. During latency, the EBV genomes remain as episomes and replicate in the S-phase. The replicated episomes are then partitioned into the daughter cells during cell division. In order to avoid host immune surveillance, only a limited repertoire of viral genes are expressed. Notably, abundant viral non-coding RNAs with low antigenicity are also synthesized to facilitate the viral replication. In response to certain physiological or pathological stimuli, the latent genome can be reactivated, characterized by a series of highly ordered events. The viral immediate-early gene products, such as Zta and Rta, will function as a molecular switch to turn on the entire lytic cascade and induce the expression of ~100 viral early and late genes. Ultimately, this leads to the production of new infectious virions [16,17].

The infectious virions can be transmitted through body fluids such as blood and urine, but they are mainly spread through saliva exchange. Like other human herpesviruses, except varicella-zoster virus (VZV), vaccine-based blockade of EBV infection is still in the development stage and not yet an option. It is estimated that ~90% of the global adult population are currently carrying EBV [8]. To date, the detailed mechanism of EBV replication in vivo is still largely unclear. Based on available limited evidence, different views of these replicative events have emerged. One view is that EBV may first enter the replication-permissive epithelial cells in the oral cavity where an active lytic replication occurs. The propagated infectious virions are then released from the initial epithelial cells to infect the nearby infiltrating B cells in which the full spectra of viral latency transcripts are synthesized (also known as type III latency) (Table 1). An alternative view is that EBV transmigrates across polarized human oral epithelial cells by apical to basolateral transcytosis without causing lytic replication and subsequently infects B cells [18]. The type III latency can activate B cells and lead to a transient expansion of the EBV-infected B cell pool. Most of infected B cells will be annihilated by the host immune surveillance. Only a subset of infected B cells can persist for the lifetime of the host. This is attributable to their expression of limited number of viral antigens. In these viral reservoir cells (i.e., memory B cells) either no (type 0 latency) or only one viral protein, EBV-encoded nuclear antigen (EBNA) 1 (type I latency) is expressed (Table 1). Furthermore, to permanently live with the hosts, new infectious virions need to be continuously produced to replenish the viral reservoir. It is believed that sporadic reactivation occurs in epithelial tissues as well as B cells differentiating into plasma cells [19,20], and the progeny virions are released to infect new host cells.

Accumulating evidence has shown that both viral latency and lytic cycle are required for EBV pathogenesis. There are approximately 100 open reading frames encoded by the EBV genome. Among them, some latent genes such as EBV-encoded nuclear antigen 1 (EBNA1) [21], EBV-encoded nuclear antigen 2 (EBNA2), EBV-encoded nuclear antigen 3C (EBNA3C), and latent membrane protein 1 (LMP1) have been shown to mediate viral oncogenesis in cell and/or animal models. These viral oncogene products can activate various tumor-associated pathways such as Notch and nuclear factor-kB (NF-kB) signalings. In addition to well-characterized viral protein-coding genes, EBV has been shown to utilize viral non-coding RNAs (ncRNAs) such as microRNAs (miRNAs), long non-coding RNAs (lncRNAs), small non-coding EBV-encoded RNAs (EBERs), as well as recently identified circular RNA (circRNA) to facilitate its life cycle and oncogenesis [22,23,24,25,26,27,28,29,30].

## 3. Presence of EBV in Lung Cancers

The human lung is not a sterile anatomic site and it is regularly in contact with the external environment [31]. A diversity of microorganisms including bacteria, yeasts, and viruses can be harbored in either healthy or diseased lung [31]. Some of those infectious microorganisms have long been speculated to contribute to lung carcinogenesis [32,33].

A possible association of EBV with LCs was first supported by the finding that some LC patients had a significantly higher level of anti-EBV IgA in the sera compared to the healthy control individuals [34]. Later on, the presence of EBV in the bronchoalveolar fluid from LC patients further suggests that the lung tissue may act as a major EBV reservoir [35]. In 1987, Begin and colleagues reported the first EBV-positive LC case [36]. In this case, a 40-year-old Asian female nonsmoker developed an uncommon type of nonkeratinizing lung squamous cell carcinoma. Histologically, this poorly differentiated epithelial tumor mimics the lymphoepithelioma-like carcinoma (LELC). In the following years, an increasing number of primary EBV-positive LELCs have been detected in the lung. Now, these subtypes of lung cancers are diagnosed as “non-small cell carcinoma, not otherwise specified” under the NSCLC group according to the 2015 World Health Organization classification [2]. These EBV-positive LELCs are characterized by poorly or undifferentiated squamoid or glandular carcinoma with intensive tumor infiltrating immune cells and preferentially occur in Asian patients [37,38,39,40].

In addition to the uncommon LELC subtype, emerging evidence has shown that EBV is also present in the tumor cells of conventional NSCLC subtypes including lung squamous-cell carcinomas (LUSC) and lung adenocarcinomas (LUAD) [41,42,43,44,45,46,47]. In addition to NSCLC, Chu and colleagues also detected EBV gene products such as EBNA1 and LMP1 in SCLC patients in the United States. However, it is unclear if the detected EBV products are from tumor-infiltrating EBV-positive (EBV(+)) B cells or the LC cells [48].

Here, we summarized the studies reporting the status and potential roles of EBV in lung carcinogenesis. Studies were identified by searching the PubMed database and only articles (including abstracts) published in English were reviewed. The latest literature search was performed in March 2021. Having carefully evaluated the extracted studies, we selected 42 relevant studies and the EBV status in LCs was summarized in Table 2 [12,36,37,38,39,40,41,42,43,44,45,46,47,48,49,50,51,52,53,54,55,56,57,58,59,60,61,62,63,64,65,66,67,68,69,70,71,72,73,74,75,76].

Having reviewed the data, we found that the association of EBV and LCs shows significant differences based on tumor histology types and geographical sites where the studies were carried out. The reported incidence rates vary from 0 to 100% (Table 2). EBV positivity was more frequently observed in Asian LC patients with the LELC subtype than patients in other racial groups or having other LC subtypes.

The variation of incidence appears also affected by the sample sizes and methodologies used for EBV infection. Notably, the majority of the studies used traditional viral screening methods such as polymerase chain reaction (PCR), in situ hybridization (ISH), and immunohistochemistry (IHC). Due to the inherent limitations of these traditional screening methods (such as PCR priming issues, usage of inappropriate/biased detection markers, inconsistent thresholds for positivity, etc.), the accuracy of the incidence data was questionable. We reasoned that an ideal detection approach should cover the full sequence of LC cells and search for any present EBV genetic materials.

To date, the next-generation sequencing (NGS) technology has been successfully utilized to discover and interrogate various oncogenic pathogens. NGS uses an unbiased method to globally assess all the exogenous microorganisms within a tumor sample with high sensitivity and specificity. Several research teams, including ours, have successfully utilized NGS (e.g., RNA-seq) to interrogate exogenous pathogens associated with human cancers [12,14,15,77,78,79,80,81,82,83,84,85]. Furthermore, NGS technology has enabled us to not only discover new oncogenic pathogens, but also identify previous false discoveries.

In addition to the traditional methods, we utilized our unbiased RNA-seq based informatics approach to comprehensively interrogate the involvement of EBV in LCs in our recent work [12]. RNA-seq datasets of 1127 LC samples were analyzed by our NGS pipeline. Samples were mainly collected from LC patients in western countries. To our knowledge, this is the largest scale of screening work for EBV infection in LCs to date. We reasoned that the sample size should be sufficient for us to draw a definitive conclusion of the involvement of EBV in LCs. Our NGS analysis showed that four LC cases exhibit transcriptional active EBV infection. In addition to NGS, we also conducted ISH to examine the expression of EBV transcripts in LC cells. Strong EBV-encoded RNA (EBER) signals were observed in three out of 110 analyzed LC tissues. Notably, the EBER signals were detected in LC cells but not in the tumor-infiltrating immune cells.

The low EBV infection rate (~0.6%) observed in our study indicates that EBV is unlikely to play a significant role in the development of LCs in the western countries, but it may contribute to the development of a subset of LC cases [12]. In areas where EBV-associated cancers are endemic, such as Southeast Asia and sub-Saharan African, the link between EBV and LCs may be more prevalent. Another rationale for the observed low EBV incidence rate is that EBV may use the hit-and-run strategy to infect lung epithelial cells, which subsequently promotes the LC development [86]. Thus, the transient presence of EBV genomes may cause genetic scars in the infected cells, leading to a permanent alteration of host gene expression and lung carcinogenesis. In accordance, Hu et al. recently provided evidence showing that EBV may use a similar hit-and-run mechanism to promote breast cancer development [87].

## 4. Etiological Role of EBV in LC

### 4.1. EBV Transcriptome

EBV exhibits various latency programs in the infected lymphoid and epithelial cells (Table 1). The type of latency program can reflect how EBV interacts with its host cells and facilitates carcinogenesis. In our recent study, we also tried to elucidate EBV latency program in LCs by conducting the first comprehensive transcriptome analysis of the EBV(+) LCs [12]. Our sequencing study detected multiple viral latency products including abundant EBNA1, LMP1, LMP2A, LMP2B, as well as BamHI A rightward transcripts (BART) in EBV(+) LC tissues, which resembles a type II-like viral latency program [12].

The high level of BART is consistent with a true EBV latency since BART is more highly expressed in the infected epithelial cells than in B cells. Although previous studies have been unable to detect protein from endogenous BART (e.g., RPMS1 and A73) [88,89], the robust expression of these transcripts indicates a functional role in LCs, presumably as lncRNAs, which has been previously proposed in the EBV(+) GC [90]. Since many known lncRNAs function in molecular complexes that inhibit transcription, it is likely that BART can selectively repress cellular gene expression in EBV(+) LCs. BART also encodes at least 44 viral intronic microRNAs (miRNAs). The pathogenic roles of these BART miRNAs in the EBV’s life cycle and in EBV-associated cancers have also been characterized [91]. The high level of BART in LCs would facilitate a significant role in modulating the cellular phenotype by BART miRNAs in LCs. Furthermore, we also observed new transcript isoforms from two novel regions within the BamHI A loci (i.e., the region between exons 4 and 5, as well as between exons 6 and 7), which are likely initiated by a hidden promoter [12]. These new transcripts may similarly play a role in non-coding RNA-mediated modulation of cellular function.

Several novel alternative splicing events of LMP2A have been detected in various EBV-associated cancers [27,92,93]. In EBV(+) LCs, the classical splicing event between the first exon (exon 1A) and exon 2 of LMP2A was not detected [12]. Instead, a novel splicing event between splicing sites located within LMP2A exon 2 and RPMS1 exon 7 was detected [12]. Thus, together with all these findings, we speculated that the alternative splicing of LMP2A may be more common than we previously expected and it may play important roles in EBV life cycle and pathogenesis.

### 4.2. EBV-Associated Immunoevasion

In some EBV-associated cancers such as NPC and GC, EBV is known to alter the tumor immune microenvironment to facilitate the carcinogenesis [15,81]. In accordance, in the context of LCs, we also observed an increased immune cell infiltration in EBV(+) LCs [12]. Despite this heightened influx of immune cells, EBV(+) LC cells persist in the patients. It suggests that EBV(+) LCs may have successfully employed certain immunoevasion strategies to ensure virus/tumor survival.

Indeed, elevated levels of multiple immune checkpoint molecules, such as indoleamine 2,4-dioxygenase (IDO), programmed cell death 1 (PD-1), cytotoxic T-lymphocyte-associated protein 4 (CTLA-4), lymphocyte-activation gene 3 (LAG3), B and T lymphocyte attenuator (BTLA), and v-domain Ig suppressor of T cell activation (VISTA) were also detected in EBV(+) LCs [12]. Further, several studies have reported that high levels of programmed death-ligand 1 (PD-L1) were observed in EBV(+) LCs [64,66]. These immune inhibitors may contribute to EBV-associated tumor immune tolerance. For example, IDO is one of the top EBV-induced immune inhibitors. IDO may inhibit the activities of cytotoxic T lymphocytes and NK cells by causing local tryptophan depletion in the tumor niche and thus enhance tumor survival [15,94,95,96].

Along this line, we also detected a high level of BNLF2a gene expression in the absence of significant expression of other viral lytic genes in the EBV(+) LCs [12]. BNLF2a is classified as an early lytic phase protein and can suppress immune detection of the EBV(+) cells by blocking viral antigen presentation to the major histocompatibility complex (MHC) class I molecules [81,97,98,99,100,101,102]. Interestingly, expression of BNLF2a has been reported in a good portion of EBV(+) GC tissues and cell lines [15,81]. Thus, it is possible that EBV infection may similarly promote LC immunoevasion by expressing BNLF2a.

### 4.3. EBV-Associated Alteration of Tumor Pathways

Unlike other EBV-associated cancers such as NPC and GC, the EBV-mediated alteration of cellular signaling pathways in lungs is poorly characterized. Having examined the cellular transcriptome of EBV(+) LCs, we found the activation of several EBV-associated oncogenic pathways and inhibition of multiple tumor suppressors [12]. It includes the alteration of G2/M and G1/S cell cycle control, the p53, HIPPO, and Sirtuin signaling pathways [12]. EBV might play a direct regulatory role in these tumor pathways. For example, the activation of breast cancer 1 (BRCA1) signaling may be triggered by EBV infection, since BRCA1 is an important signaling molecule in the innate sensing of herpesvirus DNA and EBV replication [103,104]. The activation of tumor necrosis factor receptor (TNFR) signaling in EBV(+) LCs is likely due to the expression of viral oncogene LMP1 which acts as a constitutively activated truncated form of the TNFR. Further, the activation of cyclin-dependent kinase 5 (CDK5) signaling is likely due to the expression of viral oncogene EBNA2 which enhances the expression of p35, a CDK5 activator.

### 4.4. EBV-Associated Genomic Changes

So far, the mutational landscape has only been explored in the LELC subtype of EBV(+) LCs. Emerging evidence has shown that major driver mutations for EBV-negative (EBV(−)) LCs such as epidermal growth factor receptor (EGFR), Kirsten rat sarcoma (KRAS), mesenchymal epithelial transition factor (MET), anaplastic lymphoma kinase (ALK), and c-ros oncogene 1 (ROS1) are rarely observed in the EBV(+) LCs [62,105,106,107,108]. The observation is consistent with other EBV(+) cancers such as NPC and GC which also exhibit distinct mutation profiles compared to the uninfected tumors. It is speculated that the viral oncogenes may only compensate the oncogenic effects of certain cancer driver mutations, but additional mutations are still required for cancer formation.

Recently, Hong and colleagues utilized whole exome sequencing, targeted deep sequencing and single-nucleotide polymorphism array to survey the genomic landscapes of 91 EBV(+) LC samples [68]. They found that EBV(+) LCs show distinct genomic features from EBV(−) LCs, NK/T-cell lymphoma and EBV(+) GC but share similarities with EBV(+) NPC. Furthermore, EBV(+) LCs exhibit a low degree of somatic mutation but widespread copy number variations. A widespread signature two mutations were observed in EBV(+) LCs. This is likely caused by the overactivity of the AID/APOBEC family of cytidine deaminases. The APOBEC family proteins can serve as endogenous mutagens for oncogenesis. Genetic lesions were enriched in several oncogenic pathways including NF-kB and JAK/STAT. The frequently dysregulated NF-kB signaling could be hijacked by EBV to enhance cellular survival and facilitate viral persistence. Ubiquitous losses of type I interferon (IFN) genes were also seen in EBV(+) LCs, which likely impairs the production of anti-viral cytokine and IFN-dependent JAK/STAT activation. Together, the APOBEC family gene signature, deregulated NF-kB signaling, and loss of type I IFN genes may contribute to the EBV-induced lung carcinogenesis.

Meanwhile, Chau et al. conducted a whole genome sequencing analysis of 57 EBV(+) LCs with LELC subtypes [64]. They found that EBV(+) LCs showed a unique mutation profile with low incidence of p53 mutation as well as other major cancer driver mutations in RTK/RAS/RAF and PI3K/AKT/mTOR pathways but enriched for loss-of-function mutations in several inhibitors of NF-kB pathways. Overall, the mutation pattern is different from EBV(−) LCs, but rather resembles that of EBV(+) NPC.

In another study, Chen and colleagues did whole genome sequencing analyses of eight EBV(+) LC samples with LELC subtype [66]. The somatic mutation profiles were compared to the ones of 50 EBV(−) LUAD, 50 EBV(−) LUSC, and 26 EBV(+) NPC samples. They found that EBV(+) LCs showed a total of 14 frequently mutated genes, which is much lower than those in other cancer samples analyzed. A decreased number of gene copies was also widely observed in EBV(+) LCs, including tumor-associated genes such as zinc finger and BTB domain-containing 16 (ZBTB16), peroxisome proliferator activated receptor gamma (PPARG), and transforming growth factor beta receptor 2 (TGFBR2). It is speculated that the copy number loss may be particularly important for EBV-associated lung carcinogenesis.

### 4.5. Proposed Disease Model of EBV-Associated LCs

**Figure 1:**
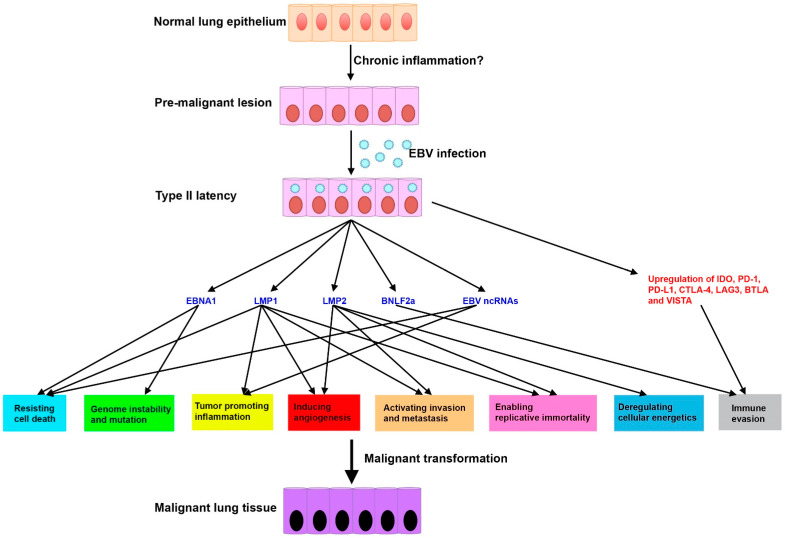
Proposed disease model of EBV-associated LCs. Some pre-existing genetic alterations induced by pro-tumor signals (e.g., chronic inflammation) in precursor dysplastic lesions are important to support EBV infection and maintain Type II latency in the lung epithelia. In the milieu of EBV-infected lesions, expression of cellular genes such as immune checkpoint molecules and viral genes including EBNA1, LMP1, LMP2, BNLF2a, and EBV ncRNAs can activate a number of cancer hallmarks (highlighted in colored box), which leads to lung carcinogenesis. Viral genes are depicted in blue and host genes in red. Activation of cancer hallmarks is predicted based on experimental data obtained from other well-established EBV-associated cancers.

In general, tumor viruses are necessary, but not sufficient for their associated cancer development. In accordance with this information, we found that primary lung epithelial cells cannot be immortalized by EBV infection alone (Lin unpublished data). Thus, in the context of EBV-associated LCs, we postulated that EBV infection only contributes to a portion of the oncogenic events. Additional genetic and epigenetic mutations and co-factors such as chronic inflammation, immunosuppression, and environmental mutagens are also required for this multistep oncogenic process. We now favor a disease model in which the impact of EBV infection on the lung carcinogenesis might be a consequence of the aberrant establishment of viral latency in lung epithelial cells that have already undergone premalignant changes (Figure 1). In our proposed disease model, some pre-existing genetic alterations induced by pro-tumor signals (e.g., chronic inflammation) in precursor dysplastic lesions are important to support EBV infection and maintain Type II latency in the lung epithelia. Persistent EBV infection induces the expression of cellular and viral genes in the milieu of infected lesions, which subsequently activates a number of cancer hallmarks [109,110] and leads to lung carcinogenesis (Figure 1). To further elucidate the etiological role of EBV, future work is warranted to screen pre-malignant lung tissues for EBV infection. The observation of monoclonal proliferation of lung epithelial cells carrying latently infected EBV will strongly indicate the presence of EBV in the early stage of lung carcinogenesis.

## 5. Experimental Models for EBV(+) LC

Understanding of the EBV-lung epithelial cell interaction depends on appropriate in vitro and in vivo experimental models. A lung cancer cell line carrying natively infected EBV should be an ideal in vitro system to explore the complex interplay between EBV and the host cells. To our knowledge, such cell systems have not yet been reported. We thus utilized our NGS pipeline to analyze the RNA-seq data sets of 182 lung cancer cell lines from the Cancer Cell Line Encyclopedia (CCLE) cohort [111], including 129 NSCLC and 53 SCLC samples. Our data showed that none of the analyzed cells carries latently infected EBV (Lin unpublished data). Since EBV(+) LC cell models are not available, we have set out to infect the lung cancer cells using the well-established cell-to-cell EBV infection method by co-culturing EBV(+) Akata cells with lung cancer cells. After EBV infection, a lung cancer cell line carrying a type II latency was successfully established (Lin unpublished data), which should be a good model to monitor the direct interaction between EBV and lung epithelial cells. Furthermore, an animal model for EBV(+) LCs has not yet been reported. Future work to develop a suitable in vivo disease model (e.g., a patient-derived xenograft EBV(+) LC mouse model) will be surely warranted and it will be critical for understanding EBV-mediated immune evasion and infection during lung carcinogenesis.

## 6. Challenges and Future Directions

One remaining puzzle is how EBV gains access to the lung epithelium when the EBV life cycle is believed to occur in oral epithelial cells and B cells. Although we cannot rule out the possibility that lung epithelium may serve as a normal reservoir for EBV infection, another possible explanation is that some inflammation events preceding lung cancer may help the EBV infection. Since lung tissue is not a sterile environment, the constant interaction between pulmonary microbiota and lung tissue may elicit inflammation that subsequently attracts lymphocytes and certain lytic-inducing epithelium-derived extracellular vesicles [112,113] to facilitate EBV infection. Further, since EBV is constantly shed into the saliva, direct aspiration of EBV(+) saliva into the lung might also be a possible route for infection.

In contrast to the well-established route for EBV entry into B cells, it is still largely unclear how EBV enters epithelial cells. We reasoned that EBV may enter the lung epithelial cells through multiple mechanisms: (1) cell-to-cell mechanism through which EBV can enter lung epithelial cells by cell membrane contact or the formation of cell-in-cell structure of EBV-infected B cells and uninfected epithelial cells [114]; (2) Cell-free virus may enter cells by directly interacting with the EBV receptor (CD21) expressed on the respiratory epithelial cells; (3) Recent studies have shown that two novel receptors neuropilin 1 (NRP1) and ephrin A2 (EFNA2) can mediate EBV entry into nasopharyngeal epithelial cells. Since NRP1 are also expressed on the lung epithelial cells, NRP1 may facilitate viral internalization and membrane fusion by interacting with EBV glycoprotein gB [115,116]. Although EFNA2 has been shown to promote the cell-free virus entry into nasopharyngeal epithelial cells, EBV is less likely to use this route since EFNA2 is only weakly expressed in lung epithelial cells [117,118].

The observation that EBV(+) LCs are mostly seen in Asian populations and that the disease is more prevalent in NPC endemic regions raises the possibility that some viral genomic variations may contribute to the unusual geographic distribution of EBV(+) LCs. As a DNA virus, EBV has long been thought to have a highly conserved genome. However, increasing lines of evidence have shown that EBV genome polymorphism may contribute to its oncogenicity. For example, Feng and colleagues reported that a particular single nucleotide polymorphism in the EBV genome (SNP G155391A) leading to an RPMS1 variant is strongly associated with NPC but not other EBV-related malignancies [119]. It will be interesting to see if this particular RPMS1 variant also impacts the development of EBV(+) LCs.

The low survival rate of LCs partially results from the fact that LCs are largely diagnosed in their late/advanced stages when surgical treatment is no longer an option. However, since EBV itself can serve as a great biomarker, routine application of EBV serology or nucleic acid testing could offer early diagnosis of EBV(+) LCs during the surgically treatable stages of this disease. Additionally, the EBV testing could also be useful in monitoring treatment and providing prognostic information. Further, since EBV(+) LCs usually do not carry the EGFR mutation, which makes conventional tyrosine kinase inhibitor (TKI)-based therapy ineffective. Thus, stratification of cancer patients with EBV(+) LCs will help choose more efficient therapeutic regimen. For example, since EBV(+) LCs usually express higher levels of PD-L1 as well as other immune checkpoint molecules, the new immune checkpoint inhibitors may help improve the overall survival of EBV(+) LCs by preventing immune evasion of the EBV-infected tumor cells. In the future, EBV(+) LC patients may also benefit from next-generation drugs targeting the EBV gene products that exclusively expressed in the cancer cells.

Together, better understanding of EBV-associated lung carcinogenesis will provide important mechanistic basis for the development of personalized cancer therapy and diagnostics that will ultimately benefit many cancer patients.

## Figures and Tables

**Table 1:** EBV gene expression in different types of latency.

Latency Types	EBV Genes	Examples of EBV Associated Cancers
0	EBER1, EBER2, RPMS1, viral miRNAs	Memory B cells in EBV(+) individuals
I	EBER1, EBER2, RPMS1, viral miRNAs, and EBNA1	Burkitt’s lymphoma
II	EBER1, EBER2, RPMS1, viral miRNAs, EBNA1, LMP1, LMP2A, and LMP2B	Nasopharyngeal carcinoma Lung cancer
III	EBER1, EBER2, RPMS1, viral miRNAs, EBNA1, EBNA2, EBNA3A, EBNA3B, EBNA3C, EBNA-LP, LMP1, LMP2A, and LMP2B	AIDS-associated lymphoma

**Table 2:** Association between EBV infection and lung cancers.

Tumor Types	Number of Total Cases	Number of EBV(+) Cases	EBV Incidence Rates (%)	Detection Methods	Geographical Sites	References
LELC	1	1	100.0	Serology	America	[36]
LELC	4	3	75.0	ISH, Serology	America	[49]
LELC	1	1	100.0	ISH, Serology, PCR	America	[50]
LELC	1	0	0.0	ISH	America	[51]
LELC	5	5	100.0	ISH, Southern Blot	Asia	[52]
NSCLC/SCLC	80	5	6.3	ISH, IHC, PCR	Asia	[44]
NSCLC	167	9	5.4	ISH, Southern Blot, IHC	Asia	[53]
LELC	1	1	100.0	ISH, Serology, PCR	Europe	[37]
LELC	11	11	100.0	ISH	Asia	[39]
LELC	2	2	100.0	ISH, PCR, IHC	Asia	[38]
LELC	2	0	0.0	ISH	Europe	[54]
NSCLC	130	0	0.0	ISH	America	[55]
LELC	1	0	0.0	IHC	America	[56]
LELC	1	1	100.0	ISH	America	[57]
NSCLC	127	11	8.7	ISH, IHC	Asia	[42]
NSCLC	5	5	100.0	ISH	Asia	[58]
LELC	1	1	100.0	ISH, PCR	Asia	[59]
NSCLC	51	30	58.8	ISH, IHC	Asia	[40]
LELC	6	0	0.0	ISH	America	[60]
LUAD	3	1	33.3	ISH, PCR, IHC	Europe	[45]
LELC	11	11	100.0	ISH, Serology, PCR	Asia	[61]
LELC	23	23	100.0	Serology	Asia	[62]
SCLC	23	1	4.3	ISH, IHC	America	[48]
LELC	1	1	100.0	ISH	Asia	[70]
NSCLC/SCLC	122	0	0.0	ISH, PCR, IHC	Europe	[63]
LELC	1	0	0.0	ISH	Asia	[76]
NSCLC	108	36	33.3	ISH	Asia	[43]
NSCLC	19	12	63.2	ISH, PCR, IHC	Europe	[41]
LUAD	110	0	0.0	ISH	Asia	[72]
NSCLC	48	7	14.6	PCR, Microarray	America	[71]
LELC	1	1	100.0	ISH, PCR, Serology	Asia	[74]
LELC	1	1	100.0	ISH	Asia	[75]
LELC	1	1	100.0	ISH	Asia	[69]
NSCLC/SCLC	48	5	10.4	PCR, IHC	Asia	[47]
NSCLC	66	4	6.1	ISH, NGS	Asia	[46]
NSCLC	1127	7	0.6	NGS, ISH	America, Europe, Asia	[12]
NSCLC	176	33	18.8	ISH, PCR	Asia	[65]
LELC	150	150	100.0	ISH	Asia	[68]
LELC	57	57	100.0	ISH	Asia	[64]
LELC	1	1	100.0	ISH, PCR	Asia, Europe	[67]
LELC	8	8	100.0	NGS	Asia	[66]
LELC	1	1	100.0	ISH	Asia	[73]

LELC: pulmonary lymphoepithelioma-like carcinoma; NSCLC: non-small cell lung cancer; SCLC: small cell lung cancer; in situ hybridization (ISH); polymerase chain reaction (PCR); immunohistochemistry (IHC); next-generation sequencing (NGS).
